# Coherent activity between brain regions that code for value is linked to the malleability of human behavior

**DOI:** 10.1038/srep43250

**Published:** 2017-02-27

**Authors:** Nicole Cooper, Danielle S. Bassett, Emily B. Falk

**Affiliations:** 1 Annenberg School for Communication, University of Pennsylvania, Philadelphia, PA, USA; 2 US Army Research Laboratory, Aberdeen Proving Ground, MD, USA; 3 Department of Bioengineering, University of Pennsylvania, Philadelphia, PA, USA; 4 Department of Electrical and Systems Engineering, University of Pennsylvania, Philadelphia, PA, USA.

## Abstract

Brain activity in medial prefrontal cortex (MPFC) during exposure to persuasive messages can predict health behavior change. This brain-behavior relationship has been linked to areas of MPFC previously associated with self-related processing; however, the mechanism underlying this relationship is unclear. We explore two components of self-related processing – self-reflection and subjective valuation – and examine coherent activity between relevant networks of brain regions during exposure to health messages encouraging exercise and discouraging sedentary behaviors. We find that objectively logged reductions in sedentary behavior in the following month are linked to functional connectivity within brain regions associated with positive valuation, but not within regions associated with self-reflection on personality traits. Furthermore, functional connectivity between valuation regions contributes additional information compared to average brain activation within single brain regions. These data support an account in which MPFC integrates the value of messages to the self during persuasive health messaging and speak to broader questions of how humans make decisions about how to behave.

Predicting human behavior is a central goal across both social and brain sciences. Recent work has demonstrated that neural measures can be used to forecast real-world, complex behavior at timescales from months to years^1^,^2^. For example, brain activity has been linked to future behavior in a number of domains, such as health behaviors^3^,^4^,^5^,^6^,^7^, response to clinical treatments^8^,^9^,^10^, learning^11^,^12^,^13^, and consumer behavior^14^,^15^,^16^. One consistent finding in this literature has been a connection between neural activity within the medial prefrontal cortex (MPFC) during persuasive messaging and subsequent behavior change. Indeed, in several domains of health behavior, higher activity in subregions of the MPFC during exposure to persuasive messaging is related to future message-consistent behavior change^3^,^4^,^5^,^7^,^17^,^18^,^19^.

Several studies have posited that the MPFC-behavior relationship stems from self-related processing. In support of this idea, health messages that are tailored to individuals, or that are rated as more self-relevant, are more likely to change attitudes, intentions and behaviors^20^,^21^,^22^,^23^. However, most studies relating MPFC activity to behavior change have not explicitly tested the self-related processing hypothesis (c.f. refs 4,19). Further, previous work has not provided a clear definition of self-related processing expected to link brain and behavior. Within the broader psychology and neuroscience literatures, self-related processing typically refers to the evaluation of one’s personality traits, physical characteristics, autobiographical memories or future goals^24^,^25^,^26^,^27^. These are largely stable, dispositional qualities that can be explicitly accessed and evaluated. It could be the case that health messaging is more likely to lead to behavior change when individuals feel that they have traits or exemplify behaviors that make them susceptible to the problems addressed in the message, increasing the self-relevance of the message. Several meta-analyses of self-related processing focus on tasks that ask participants to make judgments about their personality traits, and term this cognitive process ‘self-reflection’^28^,^29^. These studies and others have found that a subregion of the MPFC within BA10 and the posterior cingulate cortex (PCC) are most likely to be active during self-reflection judgments^28^,^29^,^30^,^31^,^32^,^33^.

However, this self-reflective aspect of self-related processing is not the only possible explanation of the relationship between MPFC activity and behavior change. Another candidate process is subjective valuation, or the value of behaviors and rewards relative to the self^17^. These values may be susceptible to influence by persuasive health messaging, such that messaging increases the positive value individuals place on the target health behavior or outcome, thereby increasing the likelihood of future message-consistent behavior change. Activity in a ventral subregion of the MPFC and the ventral striatum (VS) has been linked to value-related processing, scaling positively with subjective value across decision domains^34^,^35^,^36^,^37^.

The lack of clarity around MPFC’s specific function relative to behavior change is further complicated by the fact that it does not operate in isolation. Therefore, one way to gain new insights into the nature of the relationship between MPFC activity and behavior change is to examine the regions with which it interacts during exposure to persuasive messages using functional connectivity analysis. There is growing recognition that examining coherent activity between networks of regions provides additional, complementary information about cognition^38^,^39^,^40^,^41^,^42^,^43^. Critically, extant research has largely assessed neural activity within the MPFC alone^3^,^5^,^7^,^17^,^18^,^19^ or averaged activity across several regions within a task-relevant network^4^,^44^,^45^. Thus, we argue that investigation into both the localization of activation and functional interactions between brain regions is needed to gain a more complete understanding of brain-behavior relationships, and in particular, the role of MPFC in social influence and behavior change.

Here we test two possible accounts of the link between MPFC and later behavior change – self-reflection and subjective valuation. We examine whether functional connectivity within meta-analytically defined networks mapping onto each of these self-related processes during a health messaging intervention is associated with future message-consistent behavior change. We utilize data from a study of sedentary adults that measured neural activity with fMRI during a messaging intervention and also tracked physical activity levels before and after the intervention^19^. We find that reductions in sedentary behavior in the month after the intervention are associated with both overall activity and functional connectivity within brain regions associated with self-related valuation, but not with connectivity between regions associated with self-reflection and assessing one’s own traits. Furthermore, functional connectivity between value regions contributes additional information compared to average activation results. These data highlight a novel account of MPFC’s role in behavior change, emphasizing coherent activity in regions associated with subjective valuation, relative to the self.

## Methods

### Participants

A group of 60 sedentary adults was recruited for this study. After imaging and behavioral attrition (detailed below in Data Analysis), the final sample consisted of 44 participants (28 female; mean age = 32 years, S.D. = 13 years; 31 white). Eligibility criteria required that participants reported engaging in less than 195 minutes of moderate and vigorous physical activity per week, including walking. To establish this number, participants completed the short-form International Physical Activity Questionnaire (IPAQ). The mean number of minutes of activity per week reported at intake was 121 (S.D. = 49 min). On average, participants were overweight (mean body mass index = 27.7, S.D. = 5.5). Participants also had to meet standard fMRI eligibility criteria, including having no metal implants in their body, being right-handed, and not being pregnant or claustrophobic. Participants with a history of major health problems or mental illness were excluded. The institutional review board at the University of Michigan approved this research, and all methods were performed in accordance with the approved guidelines and the Declaration of Helsinki. All participants received written and oral instructions of the procedures, and gave written informed consent.

### Procedure

Once enrolled in the study, participants completed three appointments. At the baseline appointment, participants completed demographic surveys (including their age, gender, and ethnicity), among other measures that are not the focus of this investigation. During this appointment, an accelerometer was calibrated for each participant, and participants received instructions about how to wear and maintain the accelerometer for the duration of the study.

Participants returned an average of 10 days later (S.D. = 7 days) for an fMRI scanning session. Participants performed several tasks while inside the fMRI scanner, including the health messages intervention task. Task responses were collected with a five-button response device held in the participant’s right hand. They additionally completed pre- and post-scan self-report measures of individual differences that are not of interest in this investigation.

At the scanning session, participants were assigned to an experimental group. Half of the participants completed a values-based self-affirmation task during the fMRI scan, and half completed a control task. This manipulation is not the focus of this investigation, and experimental condition is controlled for in all models presented below. For discussion of the self-affirmation task and its effects, see ref. 19.

The final appointment took place an average of 36 days (SD = 8 days) after the scanning session. The period between the scanning session and the final appointment is referred to here as the follow-up period. Between the scanning session and the final appointment, participants received two study text messages per day via their mobile phones, repeating the messages shown during the fMRI tasks.

### Measures

#### Physical activity measurement

We collected accelerometer data during a week-long baseline period before the fMRI scan, and for approximately one month following the fMRI scan. All participants were right-handed, and wore the waterproof triaxial GENEA accelerometer on their left wrist^46^. Participants were encouraged to wear the accelerometers continuously during the baseline period and the month-long follow-up period. Triaxial accelerometer data were collected at 20 Hz and down-sampled to 1-min epochs during analysis. This resulted in a single measure of activity intensity (gravity subtracted signal vector magnitude; SVMg) per minute.

#### Health message intervention

**Figure 1:**
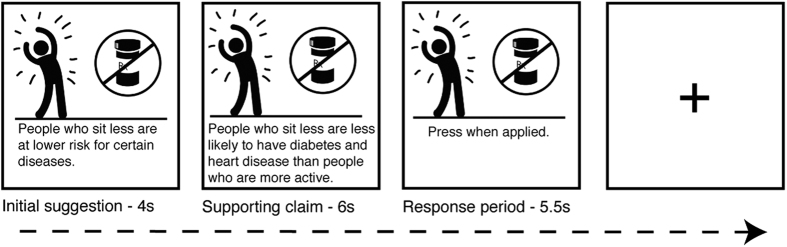
Task design. Participants viewed 40 messages promoting increased physical activity levels and reduced sedentary behavior. Each message block consisted of an initial suggestion (4 s), followed by Supporting Information (6 s). Finally, participants were asked to think about ways that they could apply this message in their own lives (response period, 5.5 s). Each message was followed by an inter-trial rest period. Credit is given to Ian Moore for image design.

The focus of the current study is 40 messages promoting increased physical activity levels and reduced sedentary behavior (physical activity trials). Messages emphasized reasons to be active or to be less sedentary (e.g., “The more you sit, the more damage it does to your body. When you sit for long periods of time, your body can’t handle sugar and fat—this can mean higher risk for disease”), and ways to accomplish these goals (e.g., “The best parking spots for your health are farther away. Choose the last row of a parking lot or the top floor so that you have farther to walk”). Each message block consisted of a short message (4 s), followed by supporting information about why participants should decrease their sedentary time or how participants could implement the suggestions, using simple text and pictograms (6 s; trial design illustrated in Fig. 1). Finally, participants were asked to think about ways that they could apply this message in their own lives and press a button for each unique application (response period, 5.5 s). The on-screen text was accompanied by audio presented through headphones. The average inter-trial interval was 1.5 seconds, with 12 second rest periods interspersed every 7^th^ block. The task included two additional categories of messages that were not the focus of the current analysis. These were a set of 10 explicitly risk-focused messages and a set of 20 messages containing information about other daily activities unrelated to sedentary behavior.

### Data Analysis

#### Imaging data acquisition and analysis

fMRI data were acquired using a 3-T GE Signa MRI scanner. A spoiled gradient echo sequence (SPGR) was used to acquire high-resolution T1-weighted structural images (124 slices; slice thickness, 1.02 mm × 1.02 mm × 1.2 mm). A reverse spiral sequence was used to acquire functional images (43 axial slices; field of view, 220 mm; slice thickness, 3 mm; voxel size, 3.44 mm × 3.4 mm × 3.0 mm repetition time, 2,000 ms; echo time, 30 ms; flip angle, 90°;). In-plane T1-weighted overlay images were also acquired (43 slices; slice thickness, 3 mm; voxel size, 0.86 mm × 0.86 mm × 3.0 mm), allowing two-stage co-registration. The first five volumes (10 s) of each run were discarded before analysis to allow for the stabilization of the BOLD signal. Functional data were preprocessed using the Statistical Parametric Mapping package (SPM8; Wellcome Department of Cognitive Neurology, Institute of Neurology, London, UK) for all steps other than initial despiking, which was performed with the 3d Despike program in the AFNI toolbox. A sinc interpolation algorithm was used to correct for differences in time of acquisition across the 43 slices with the first slice as a reference. Motion artifacts were corrected by spatial realignment to each volume’s first slice. The mean image across all functional images and the high-resolution T1 SPGR image were coregistered to the in-plane T1 image. The high-resolution T1 images were then segmented into white and gray matter and the skull was removed. Structural and functional images were normalized to the skull-stripped MNI template (“MNI152_T1_1 mm_brain.nii” from the FSL software package). Finally, the functional images were smoothed using an 8 mm FWHM Gaussian kernel.

#### Accelerometer data processing

A trained research assistant who was blind to study condition visually inspected the accelerometer data, and tagged windows of likely nonwear (e.g., extended flat lines of no activity) and sleep (e.g., contiguous low activity overnight). The remaining time during which participants were awake and wearing the device was used in further analysis. Data were excluded by day if the participant wore the device for less than 4 hours; of 1,772 person days tagged, 1,668 days, or 94%, were eligible for analysis.

Sedentary behavior was calibrated for each participant at the baseline appointment. Participants performed at least 30 min of sedentary activity (completing surveys while seated at a computer) during the baseline appointment, and the peak acceleration during this period was used to determine the appropriate cut points for sedentary time during analysis. These sedentary cut points were used in further analysis of the accelerometer data to compute the percent of each day that participants were sedentary. The use of triaxial accelerometers calibrated using specific activities completed in the laboratory has been previously validated^47^,^48^,^49^. The baseline sedentary percentage was determined by averaging across the full one-week baseline period. The post-scan sedentary percentage was the average across the one-month follow-up period.

#### Regions of interest

**Figure 2:**
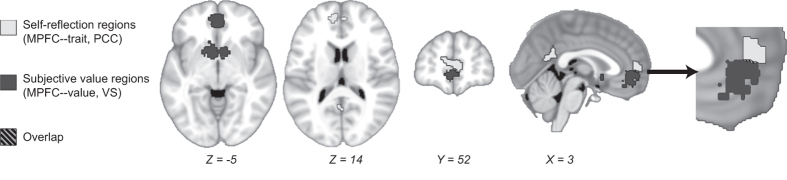
Regions of interest. Self-reflection (MPFC-trait and PCC, white) and subjective valuation (MPFC-value and VS, black) regions are displayed. The far right panel demonstrates the slight overlap between MPFC-trait and MPFC-value (hatched grey).

To address the study aims concerning the self-related processes of self-reflection and subjective valuation, we selected two regions of interest (ROIs) strongly associated with each of these processes (displayed in Fig. 2). ROIs for self-reflection were defined based on a meta-analysis of 25 studies^29^, which identified the posterior cingulate cortex (PCC, volume = 2.22 cm^3^) and a subregion of medial prefrontal cortex (MPFC-trait, volume = 2.54 cm^3^) as most active during personality judgments about oneself as compared to a semantic control condition.

ROIs for valuation were taken from a quantitative meta-analysis of 206 studies that reported subjective value-related neural signals during decision-making^34^. This meta-analysis identified the ventral striatum (VS, volume = 4.00 cm^3^) and a subregion of medial prefrontal cortex (MPFC-value, volume = 3.58 cm^3^) as most likely to be active during personal value-related decision-making. The regions used here are reported in Fig. 9 of that paper, and are the conjunction of several valuation-relevant contrasts. The study authors provided masks of the meta-analysis results. The MPFC ROIs overlap minimally (volume of intersection = 0.016 cm^3^).

#### Health message task analysis

Participants completed two functional runs of the health messages task (308 volumes each; 616 volumes total). For the first six participants, a slightly longer version of the task was used (3 functional runs of 257 volumes each, 771 volumes total). Fixed-effects models were constructed in SPM8 for each participant that included regressors for each of the 5 message periods (physical activity trials and their response periods, daily activity trials and their response periods, and risk trials). Six rigid-body movement parameters derived from spatial realignment were included as nuisance regressors in all first-level models. Data were highpass filtered with a cutoff of 128 s. The first-level contrast images were combined using a random effects model. Univariate activity estimates were extracted from each of the four regions of interest using MarsBaR^50^ and averaged across all voxels within each ROI. Activity estimates were converted to percent signal change by dividing parameter estimates by the constant for each participant.

#### Psychophysiological interactions

We used psychophysiological interactions (PPIs)^51^ to test the functional relationships between our regions of interest during message exposure. Analysis of psychophysiological interactions examines whether neural activity depends on an interaction between task conditions and activity in a specified seed region of interest. We used two MPFC subregions (described above) as seed regions, in separate models. This analysis utilized the SPM generalized PPI toolbox^52^. First-level PPI models included two PPI regressors – physical activity trials and their response periods. As is the default in the generalized PPI toolbox, the PPI regressors were created in the following way for each participant: (1) the time series of the seed region was defined as the first eigenvariate of BOLD activity in all voxels within the seed region (adjusted for effects of interest); (2) the resulting time series was deconvolved with the canonical HRF using the deconvolution algorithm in SPM8^53^; (3) the deconvolved time series was multiplied by the predicted, pre-convolved time series of each condition, which resulted in one “neural” PPI for each regressor; and (4) each neural PPI was convolved with the canonical HRF. As covariates of no interest, the PPI models included all regressors described above in the standard first-level models (physical activity trials and response periods, daily activity trials and response periods, risk trials, and the 6 motion parameters) and the time series of the seed region. To investigate group-level PPI effects, the first-level contrast images were combined using a random effects model. Using MarsBaR, average parameter estimates of functional connectivity (from target MPFC regions to matched PCC or VS ROIs) were extracted at the group level.

#### Associations with behavior change

We tested the relationship between changes in sedentary behavior and functional connectivity during message exposure in several models. The dependent variable in the first group of models was the change between the average percentage of the day that participants spent sedentary during the pre-scan baseline period and the average sedentary percentage during the post-scan follow-up period (equations 1, 2). A negative change represents a reduction in sedentary time (i.e., message consistent behavior). Psychophysiological interactions between the self-reflection regions (referred to as PPI_MPFC-trait→PCC_) and value regions (referred to as PPI_MPFC-value→VS_) were included in separate models as predictors of sedentary behavior change. All models controlled for experimental condition (affirmation *vs*. control) and demographic variables, consisting of gender, centered age, and ethnicity (white *vs*. other).


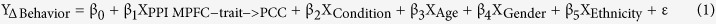



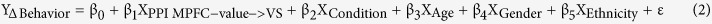


An exploratory whole-brain analysis is reported in Table 1. Multiple regression was used to predict whole-brain PPI maps from behavior change (equations 3, 4), controlling for experimental condition (affirmation *vs*. control) and demographic variables (gender, centered age, ethnicity (white *vs*. other)). The resulting image map was thresholded at *p* < 0.005 and cluster corrected with AFNI’s 3d ClustSim to *p* < 0.05 (*k* = 125 for self-reflection PPI, *k* = 133 for value PPI).


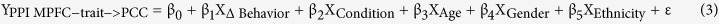



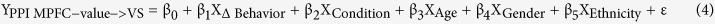


Next we examined changes in post-scan sedentary behavior using hierarchical linear models (equations 5, 6, 7). The dependent variable in these models was the percentage of the day that participants spent sedentary on each day of the follow-up period. Each model included a PPI term (PPI_MPFC-trait→PCC_ or PPI_MPFC-value→VS_) and the averaged univariate activity from matched ROI pairs (MPFC-trait and PCC [“AvgAct(MPFCt + PCC)”], or MPFC-value and VS[“AvgAct(MPFCv + VS)”]) as predictors of interest. Additional predictor variables were the post-scan date (index 1–30, or the last date on which data was collected for each participant [“DailyPostSedTime”]), centered average baseline levels of sedentary behavior (“PreSedTime”), experimental condition (affirmation *vs*. control), and demographic variables (gender, centered age, and ethnicity). The interaction term between the averaged univariate activity predictor and the post-scan date was also included as a predictor of interest. All time series mixed-effects models account for non-independence of data within participants using the lmer package in R (version 0.98.945).


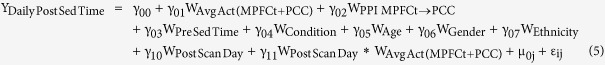



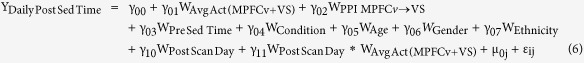


For each PPI region pairing, three versions of this model were run, varying the univariate activity predictor. The primary model included the averaged univariate activity from matched ROI pairs (as described above). In the remaining models, the univariate activity in MPFC (MPFC-trait or MPFC-value) or the univariate activity in the target region (PCC or VS) was substituted for the averaged univariate activity across regions.

To test whether the PPI_MPFC-value→VS_ variable was a significant contributor to the model predicting behavior change, ANOVA was used to compare model fits of the primary hierarchical model described above and a model reduced by the removal of the PPI term.

To test whether sedentary behavior decreases across the post-scan period, irrespective of brain activity, we used a modified version of equation 6 without any neural regressors:


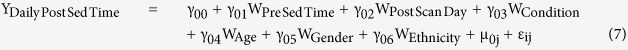


#### Imaging and behavioral data attrition

Sixty-one participants completed all appointments, including the fMRI scan. A total of 13 participants had accelerometer difficulties resulting in data loss. Data from 3 participants were lost due to excessive movement or technical problems during fMRI scanning. One additional participant was excluded from analysis because their age was outside of recruitment criteria (77 years, >3 standard deviations above the mean). Primary results are not substantially affected by the inclusion of this participant.

This resulted in a final sample of 44 included participants with neuroimaging, accelerometer, and demographic data present. The functional connectivity metric between MPFC-trait and PCC contained one outlier (>3 standard deviations from the mean), as did average activation in MPFC-trait. The outlier participants are excluded from analyses involving those terms as applicable.

## Results

### Functional connectivity between valuation regions is associated with reductions in sedentary behavior

We examined whether functional connectivity during message exposure in self-reflection and value regions was associated with changes in sedentary behavior in the month following the physical activity intervention. The mean daily sedentary time in the pre-scan week was 49.91% (SD = 13.57%), and the mean daily sedentary time in the post-scan month was 48.40% (SD = 15.70%). Although the average of participants’ sedentary time during the post-scan period is not significantly reduced (paired *t*(43) = 1.04, *p* = 0.30), there is a significant decline during the post-scan month across participants in the daily time that participants spent sedentary (*t*(37) = −3.86, *p* = 1.2 × 10^−4^; see equation 7).

**Figure 3:**
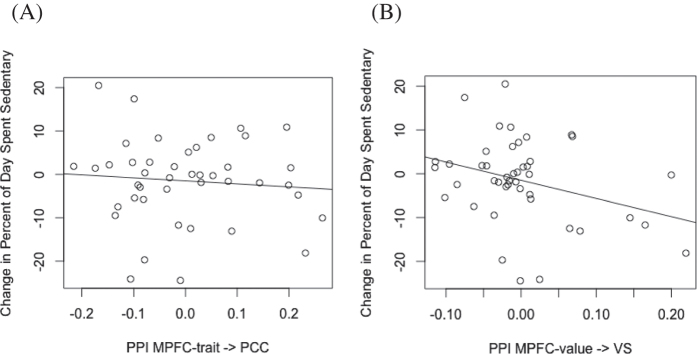
Functional connectivity between valuation regions is associated with behavior change. Changes in sedentary behavior measured one month after the messaging intervention are plotted against (**A**) functional connectivity within self-reflection regions (PPI MPFC-trait ⇒ PCC) during messaging as compared to rest and (**B**) functional connectivity within valuation regions (PPI MPFC-value ⇒ VS) during messaging as compared to rest. A negative change corresponds to a reduction in sedentary time.

Using analyses of psychophysiological interactions (PPIs), we compared the levels of functional connectivity in self-reflection and value regions during message exposure and during rest. We separately tested functional connectivity between self-reflection regions (PPI_MPFC-trait→PCC_) and value regions (PPI_MPFC-value→VS_), using these PPI estimates as predictors of changes in sedentary behavior between the pre- and post-scan periods. These models controlled for experimental condition (affirmation *vs*. control) and demographic variables (see equations 1, 2). We found that PPI within self-reflection regions (PPI _MPFC-trait→PCC_) was not significantly related to lowered sedentary time (*t*(37) = −0.56, *p* = 0.58). However, PPI within value regions (PPI _MPFC-value→VS_) was significantly related to reductions in sedentary time (*t*(38) = −2.18, *p* = 0.036), such that those participants with higher connectivity between value regions during message exposure were more likely to reduce the time that they spent being sedentary in the month following the scan. These relationships are displayed in Fig. 3. Functional connectivity is plotted against changes in sedentary behavior, controlling for experimental condition and demographics. A negative change represents a reduction in sedentary time.

Although affirmation condition is controlled for in all models presented, we also tested whether it was associated with connectivity in our regions of interest. Affirmation condition was marginally associated with connectivity between the MPFC-trait and PCC ROIs (*t*(41) = −1.75, *p* = 0.09) but not connectivity between the MPFC-value and VS ROIs (*t*(42) = −1.47, *p* = 0.15). There was not a significant interaction between affirmation condition and connectivity in self-reflection regions (*t*(36) = 0.87, *p* = 0.39) or connectivity in value regions (*t*(37) = 0.22, *p* = 0.83) relating to behavior change.

To determine whether connectivity from MPFC to additional regions outside of our target regions of interest was associated with behavior change, we ran two exploratory whole-brain searches for regions whose connectivity with the MPFC seed regions of interest was related to subsequent behavior change (see equations 3, 4 for regression models). Additional regions in which increased connectivity with MPFC-trait was correlated with larger reductions in sedentary time included the inferior temporal gyrus and the amygdala (see Table 1 for full results). No significant effects at the whole-brain level were observed for the MPFC-value ROI seed.

### Functional connectivity provides additional information compared to univariate activity

Previous work linking the MPFC and behavior change has examined overall activity in MPFC, rather than its level of functional connectivity with other regions. We performed additional analyses to examine whether overall univariate activity levels and functional connectivity were both associated with behavior change. A previously published analysis of this dataset reported an interaction between activity in a subregion of MPFC (defined by^3^ as related to behavior change) and time (post-scan day), such that participants who demonstrated higher activity in MPFC during message exposure had a steeper decline in their sedentary behavior in the month following the scan^19^. The subregion of MPFC used in that paper overlaps with the value-related subregion described here, but not with the trait self-reflection subregion.

We performed a similar analysis in this dataset. Hierarchical linear models used the percentage of each day spent sedentary during the follow-up period (post-scan) as the dependent variable (see equations 5, 6). The predictors of interest were a PPI term (PPI_MPFC-trait→PCC_ or PPI_MPFC-value→VS_); post-scan day (index 1–30, or to the last date on which data was collected for each participant); and univariate activity from the paired ROIs. As in Falk *et al*.^19^, we tested the interaction between post-scan day and univariate activity, to determine whether higher univariate activity was associated with steeper declines in sedentary behavior over the follow-up period.

In this model, we found that PPI _MPFC-trait→PCC_ was again not significantly related to post-scan sedentary time (*t*(33) = −0.55, *p* = 0.59) when univariate activity was included in the regression model. Averaged univariate activity in MPFC-trait and PCC was not related to steeper declines in sedentary behavior (*t*(33) = −0.88, *p* = 0.38). When examined in separate models, the time by activity interaction was not significantly related to behavior in either ROI (MPFC-trait *t*(32) = −1.16, *p* = 0.25; PCC, *t*(33) = −1.24, *p* = 0.22). These results suggest that neural correlates of self-reflection as specified in the Murray *et al*.^29^ meta-analysis do not mediate the relationship between MPFC activity and behavior change in this context.

By contrast, PPI _MPFC-value→VS_ did remain significantly related to reduced post-scan sedentary time (*t*(34) = −2.17, *p* = 0.036) when corresponding univariate activity was included in the model. In addition, the interaction between post-scan day and averaged univariate activity in MPFC-value and VS was significantly related to steeper reductions in sedentary behavior (*t*(34) = −2.80, *p* = 0.005). When examined in separate regressions, the time by activity interaction was significantly related to behavior in each ROI, with a particularly strong effect in VS (MPFC-value, *t*(34) = −1.99, *p* = 0.047; VS, *t*(34) = −3.7, *p* = 2.3 × 10^−4^).

To formally test whether PPI _MPFC-value→VS_ significantly improved the fit of this model, we compared model fits between the full model and a model reduced by the removal of the PPI _MPFC-value→VS_ term. The full model including PPI _MPFC-value→VS_ was a significant improvement compared to the reduced model containing only averaged univariate activity and covariates (χ^2^(1,N = 44) = 5.38, *p* = 0.02). Functional connectivity between valuation regions therefore provides additional information about future behavior change compared to average activation.

## Discussion

Recent studies have demonstrated that neural activity in the MPFC during exposure to persuasive messaging is associated with future message-consistent behavior change, and have posited that this relationship is due to self-related processing^3^,^4^,^7^,^17^,^18^,^19^. Here we distinguished two possible dimensions of self-related processing, self-reflection and subjective valuation (i.e., value of inputs to the self), and examined the functional interactions within networks of brain regions representative of these two processes. The fast-growing study of brain networks has made clear that brain regions do not operate in isolation; rather, networks of brain regions interact dynamically to produce cognitions and behaviors^39^,^40^,^41^,^42^,^43^. This approach has not previously been applied to the question of how the MPFC functions during exposure to persuasive messaging, or why this activity might be linked to behavior change.

Our data demonstrated that functional connectivity within a subjective valuation network was significantly related to changes in sedentary behavior following a physical activity messaging intervention. Participants who had higher levels of connectivity between a value-related, ventral subregion of MPFC and VS during health messages as compared to rest were those who showed a greater reduction in objectively measured sedentary time in the month following the scan. Greater averaged activation in these regions (and activation in each region individually) was also related to a steeper decline in sedentary time. Crucially, the functional connectivity and overall activation metrics in value regions were independently associated with behavior change, confirming that these metrics do not simply represent the same information.

We suggest that health messaging may increase the value that participants place on message-related behaviors or outcomes, in this case physical activity and its associated health benefits, which are then associated with increased physical activity in the month following the intervention. In the neuroeconomics literature, value-related activity in these regions has been shown to reflect changes in preferences, for example as a result of exposure to the opinions of others^14^,^54^,^55^,^56^. We find a particularly strong relationship to future behavior in VS activation, which is central to reward-based learning, motivation, and affect (for reviews, see ref 57, 58, 59). This result highlights the potential role of message value in how humans make decisions about changes in their behavior, and motivates further investigation into the function of valuation more broadly in persuasion.

This novel demonstration that functional connectivity is significantly related to behavior even controlling for activation in these regions is consistent with the idea that the integration of information about value in relation to the self is an important factor in observed behavior change. Several studies have reported increased functional connectivity between ventral MPFC and VS during processing of feedback in learning tasks^60^,^61^,^62^. More broadly, the MPFC has been identified as a key region for numerous functions and as an information processing hub^63^,^64^,^65^,^66^; as such, the MPFC can connect systems throughout the brain to integrate conceptual and affective information and to guide behavior^27^,^67^. Our finding, then, could be a signature of the integration of the increased value participants place on physical activity into their perceptions about themselves and their beliefs and goals, which contributes to ongoing reductions in sedentary behavior.

We did not find a relationship between behavior change and functional connectivity within a self-reflection network (MPFC and PCC), suggesting that at least in this context, the uptake of message information and later behavior change may rely less on integrating information about dispositional traits through connectivity between these regions (c.f., ref. 17, which finds that univariate activity in a different, functionally-defined self-reflection region of MPFC is associated with message consistent behavior change). The exploratory analysis of functional connectivity from MPFC-trait to the whole brain found that greater connectivity with regions including the amygdala, parahippocampal gyrus, and inferior temporal gyrus was related to greater behavior change. This could be an indication of the importance of other specific elements of self-related processing. For example, connectivity between MPFC and amygdala could index message emotion or salience^68^,^69^,^70^, such that those individuals who find the message more salient or emotional and self-relevant are more likely to increase their message-consistent behavior. Likewise, connectivity between MPFC and the parahippocampal gyrus, involved in prospection and memory^63^,^71^,^72^,^73^, might indicate that individuals for whom the messages are in line with personal memories or goals may be more likely to change their behavior. Further work could confirm and explore the relative influence of these additional components of self-related processing on behavior change.

Future research may also explore the robustness of these results in different psychological contexts. For example, in addition to the focal health message trial types, participants in the current study were exposed to messages about health risks from physical inactivity (N = 10) and about daily behaviors unrelated to physical inactivity (N = 20), which could have influenced participants’ neural and psychological responses to the target aspects of the task. Future work designed to address interactions between individual differences in functional connectivity and particular types of messaging will advance our understanding of potential boundary conditions on the effects observed.

In summary, in this report we investigated the relationship between neural responses in subregions of MPFC during message exposure and later message-consistent behavior change, with the goal of understanding the function of MPFC during persuasive messaging. Our results indicate that activity in and functional associations between MPFC and VS, key regions in value-related processing, are related to future behavior change. These results highlight the importance of the integration of information about the value of message content during health messaging, and emphasize the importance of considering message value in theories of persuasion and behavior change. Our novel demonstration that a measure of brain dynamics is linked to future behavior motivates increased collaboration across social and brain sciences. Understanding functional interactions between key brain regions during messaging will provide new information about what makes messages effective, and in turn improve message design as well as contribute to theories of persuasion and behavior change. Further utilization of this combination of methods would improve our understanding of how humans make long-term decisions about their behavior, and could have a large impact in applied areas such as the design of public health campaigns.

## Additional Information

**How to cite this article:** Cooper, N. *et al*. Coherent activity between brain regions that code for value is linked to the malleability of human behavior. *Sci. Rep.* **7**, 43250; doi: 10.1038/srep43250 (2017).

**Publisher's note:** Springer Nature remains neutral with regard to jurisdictional claims in published maps and institutional affiliations.

## Figures and Tables

**Table 1:** Exploratory whole-brain analysis relating behavior change to psychophysiological interactions with MPFC-trait seed.

Cluster Size	Peak T	x,y,z {mm}	Laterality	Region Label
238	−5.07	25 8 −20	R	Temporal pole
	−4.54	32 −2 −26		Amygdala
	−3.6	22 −19 −47		Fusiform gyrus
235	−4.19	53 −47 −20	R	Inferior temporal gyrus
	−3.52	53 −19 −29		Fusiform gyrus
241	−4.16	−61 −33 −20	L	Inferior temporal gyrus
	−3.79	−20 −19 −44		Fusiform gyrus
135	−3.88	−30 1 58	L	Middle frontal gyrus

A negative relationship indicates that stronger connectivity relates to a larger decrease in sedentary time.
